# Inbreeding depression affects the growth of seedlings of an African timber species with a mixed mating reproductive system, *Pericopsis elata* (Harms) Meeuwen

**DOI:** 10.1038/s41437-024-00709-x

**Published:** 2024-08-01

**Authors:** Dieu - Merci Assumani Angbonda, Crispin M. Ilunga-Mulala, Nils Bourland, Hans Beeckman, Faustin Boyemba, Hulda Hatakiwe, Jean Pierre Ngongo, Olivier J. Hardy

**Affiliations:** 1 Faculté de Gestion des Ressources Naturelles Renouvelables, Université de Kisangani, Kisangani, Democratic Republic of the Congo; 2 Evolutionary Biology and Ecology Unit CP 160/12, Faculté des Sciences, Université Libre de Bruxelles, Brussels, Belgium; 3 Laboratoire d’Ecologie et Aménagement Forestier (LECAFOR), Université de Kisangani, Kisangani, Democratic Republic of the Congo; 4 Institut National Pour l’Etude et la Recherche Agronomiques de Yangambi (INERA – Yangambi), Yangambi, Democratic Republic of the Congo; 5 Forest Is Life, TERRA Teaching and Research Centre, Gembloux Agro-Bio Tech, University of Liège, Gembloux, Belgium; 6 Faculté des Sciences, Université du Cinquantenaire de Lwiro, Lwiro, Democratic Republic of the Congo; 7 Service of Wood Biology, Royal Museum for Central Africa, Tervuren, Belgium; 8 Faculté des Sciences, Université de Kisangani, Kisangani, Democratic Republic of the Congo; 9 Center for International Forestry Research (CIFOR), Kisangani, Democratic Republic of the Congo; 10 Department of Natural and Renewable Resources Management, Faculty of Agricultural Sciences, University of Kindu, Kindu, Democratic Republic of the Congo

**Keywords:** Inbreeding, Consanguinity

## Abstract

Selfing or mating between related individuals can lead to inbreeding depression (ID), which can influence the survival, growth and evolution of populations of tree species. As selective logging involves a decrease in the density of congeneric partners, it could lead to increasing biparental inbreeding or self-fertilization, exposing the population to higher ID. We assessed the influence of inbreeding on the growth of a commercial timber species, *Pericopsis elata* (Fabaceae), which produced about 54% of self-fertilized seedlings in a natural population of the Congo basin. We followed the survival and growth of 540 plants raised in a plantation along a gradient of plant density (0.07–15.9 plants per m^2^). Parentage analysis allowed us distinguishing selfed and outcrossed seedlings. The annual growth was higher for outcrossed than selfed plants, on average by 10.8% for diameter and 12.9% for height growth. Based on the difference in above ground biomass between selfed and outcrossed seedlings after 41 months, we estimated the level of ID at *δ* = 0.33, while a lifetime estimate of ID based on the proportions of selfed plants at seedling and adult stages led to *δ* = 0.7. The level of ID on growth rate did not change significantly with age but tended to vanish under high competition. *Pericopsis elata* is a particularly interesting model because inbreeding depression is partial, with about 26% of reproducing adults resulting from selfing, contrary to most tropical tree species where selfed individuals usually die before reaching adulthood. Hence, the risks of ID must be considered in the management and conservation of the species.

## Introduction

Inbreeding depression (ID) is the reduction in fitness (*W*) of inbred offspring compared to noninbred ones due to increased homozygosity, and is often quantified by *δ* = 1 – *W*_inbred_/*W*_noninbred_. Most ID results from the accumulation in the genome of deleterious recessive or partially recessive mutations that are maintained at low frequency in populations because their deleterious effect is not or minimally expressed in the heterozygous state (Crnokrak and Roff 1999; Charlesworth and Willis 2009). This phenomenon can occur following the mating between closely related individuals or, in self-compatible hermaphrodites, like many plant species, following self-fertilization (autogamy or geitonogamy). ID affects most sexual organisms, including hermaphroditic organisms (Uyenoyama et al. 1993; Charlesworth 1987; Crnokrak and Roff 1999 ; Keller and Waller 2002). Its severity may be sensitive to the way inbreeding occurs (Wang 2000; Day et al. 2003; Glémin et al. 2003; Kristensen et al. 2003). Species or populations with an excess of consanguineous mating will express and purge more efficiently (partially) recessive deleterious mutations, eventually reducing the intensity of ID, although they may still accumulate a genetic load due to the fixation of moderately deleterious mutations (Charlesworth and Charlesworth 1998), (Therefore, ID is thought to be a key factor affecting the evolution of the mating system (Schemske and Lande 1985; Charlesworth 1987; Kristensen et al. 2003; Husband and Schemske 1996). Many theoretical works have questioned the origin and maintenance of a mixed reproductive system (i.e., coexistence of selfing and outcrossing) because the simplest models suggest that only complete outcrossing or complete self-fertilization are evolutionarily stable strategies (Holsinger 2002). Nevertheless, nearly 40% of plant species maintain a mixed mating system (selfing rate between 20 and 80%; Goodwillie et al. 2010) and these species seem to display as much ID as outcrossed species (Winn et al. 2011a).

ID is a major concern in conservation genetics because it is one of the main factors involved in the vortex of extinction following a reduction of population size, potentially bringing populations of threatened species at risk of extinction (Frankham 2005; Frankham et al. 2002; Spielman et al. 2004). The level of ID (*δ*) varies among species but also among populations or lineages, and according to environmental conditions. Meta analyses show that ID is usually stronger under stressful conditions (Reed et al. 2002; Fox and Reed 2010; Sandner et al. 2021), so that it could be minimized under experimental conditions even when it is strong in the wild, but exceptions are common, including cases where ID is stronger under optimal conditions for growth and reproduction (Armbruster and Reed 2005; Rehling et al. 2019; Sandner et al. 2022; Sandner and Matthies 2016). To assess how ID can affect the future of threatened species, it is therefore of interest to measure ID under a range of environmental conditions, including different levels of stress, and for different populations, lineages or families.

In plants, ID has most often been studied on annual or perennial herbs, which are easier to manipulate than trees for example, for controlling mating or estimating lifetime reproductive success. The deficit of experimental studies on ID in trees is particularly important for tropical tree species. In fact, most tropical tree species have developed strategies to avoid inbreeding and, in the case of hermaphroditic species, strategies to avoid self-fertilization (Dick et al. 2008). In self-compatible tropical trees the selfing rate is generally low, typically <5% (e.g. Lourmas et al. 2007), sometimes 10–30% (e.g. Duminil et al. 2016a; Hardy et al. 2019), and inbreeding detectable at the seed or seedling stage (heterozygote deficiency relative to Hardy-Weinberg equilibrium) usually disappears at the adult stage, indicating that self-fertilized or other inbred individuals have not survived to the adult stage (e.g. Hardy et al. 2019). However, there are exceptions, with some species having a truly mixed-mating system, like some *Eucalyptus* species (Byrne 2008). In tropical Africa, a notable exception is *Pericopsis elata* (Harms) Meeuwen, a large legume tree from the dense forests, in which a selfing rate of 55% has been measured in seeds and young seedlings in a population from DRC (Angbonda et al. 2021). In the studied population, the proportion of selfed adults dropped to about 26%, probably reflecting the impact of ID on survival rate. However, contrary to most self-compatible tropical tree species, ID would not be total (i.e. *δ* < 1) in *P. elata* as a non-negligible proportion of selfed seedlings reach adulthood in the population.

*Pericopsis elata* (Fabaceae) is a large upper canopy tree of the African rainforest, highly traded in the Congo Basin due to its excellent wood quality (Bourland et al. 2012a). It is called by the trade names Afrormosia or Assamela, or sometimes “African teak” because it is often used as a substitute for teak. The species currently suffers from a regeneration deficit in the wild, so that it is threatened by overexploitation linked to logging activities (Bourland et al. 2012a). The resulting reduction in the density of reproductive trees compromises the sustainability of its exploitation throughout its range. In addition to international export pressures, habitat degradation and insufficient regeneration issues are causing some of its populations to decline, and currently the species is rarely found in dense populations although locally we can find a fairly high density (Dickson et al. 2005). Therefore, *P. elata* has been included in the IUCN Red List of Threatened Species (http://www.iucnredlist.org) and in Appendix II of the Convention on International Trade of Endangered Species (CITES). Managers of tropical forests are increasingly interested in making use of *P. elata* seedings in plantations as a nature-based solution (Seddon et al. 2020) to achieve conservation goals and possibly address climate change mitigation challenges (Griscom et al. 2017; Ilunga-Mulala et al. 2024). Such successful and cost-effective plantings require proper silvicultural methods (Ilunga-Mulala et al. 2024), determining an optimal plantation density, and a good knowledge of the ecology of the species and its reproduction cycle, especially since we discovered that it is prone to high inbreeding and probably to ID. Seed quality is primordial for plantation operations, so that estimating ID, assessing how it varies according to environmental conditions, intra-specific competition (stress), and families, and assessing whether higher quality seeds could be obtained from seed trees less prone to selfing are important knowledge.

This study aims to analyze the effects of inbreeding on the growth and survival of *P. elata* seedlings raised in a nursery (for 9 months) and transplanted in an experimental setup with a gradient of plant density implying a gradient of intra-specific competition (Nelder design; 4 years of growth). We address the following questions: (1) What is the rate of selfing and does it vary among mother trees? (2) Does inbreeding affect seedling survival and/or sapling growth rate? (3) How important is the impact of inbreeding depression on growth compared to the impact of plantation density or of the maternal genotype? (4) What is the level of inbreeding depression using above-ground biomass accumulation of saplings as a proxy of fitness, or using the decay in the proportion of selfed individuals that survive until adulthood (lifetime ID)? (5) Does the impact of inbreeding depression on seedling growth vary according to (i) the age of plants, (ii) the level of intra-specific competition (density), and/or (iii) the maternal origin of plants? Finally, we discuss the implications of our results for the management of *P. elata* and plantations.

## Materials and methods

### Study species

*Pericopsis elata* is an emblematic tree species of tropical rainforest from West and Central Africa. It can reach 1.7 m in diameter at breast height (dbh) with a total height of fully developed trees varying between 30 and 60 m. It is indicative of disturbances (Boyemba Bosela 2011; Bourland et al. 2015), and is either ranked among the pioneer or non-pioneer light-demanding species, depending on the authors (Ampofo and Lawson 1972; Hawthorne 1995; Veenendaal et al. 1996; Kyereh et al. 1999). The minimum dbh of fertility is 32.3 cm and the mortality rate (recorded on 137 adult stems over a 30-year period) is 0.85% in eastern Cameroon (Bourland et al. 2012b). Its 15 mm long and 13–14 mm wide flowers are hermaphrodite and stand in short terminal panicles on a thin, hairy white rachis (Boyemba Bosela 2011). Its fruits are green to brown pods, containing 1 to 5 discoid seeds (Dickson et al. 2005), dispersed by wind (Hawthorne 1995), usually <100 m from the mother tree (Angbonda et al. 2021). The seed is almost rectangular and about 15 mm wide (Toussaint et al. 1953). Seed germination is rapid, sometimes even in the deep shade of the forest and in small forests, but seedling development remains dependent on gaps or other openings. In general, germination occurs between 6 to 12 days under controlled conditions, at a high rate. Ouédraogo et al. (2014) and Umunay et al. (2017) demonstrated that seedlings in large canopy gaps (from 50 m × 50 m) were more likely to survive than under closed canopy conditions, though more opening might be needed to reach optimal growth and survival dynamics.

### Sampling

#### Study site and sampling

To conduct our experiment, seeds of *P. elata* were collected in the Biaro natural forest (00° 12 ‘N, 25° 20’ E), located ~38 km south of Kisangani at an average elevation of 435 m, Tshopo province, Democratic Republic of Congo (DRC). It is a minimally disturbed forest that has never been logged and has been granted full conservation status by the Ministry of the Environment (Lomba 2011). All *P. elata* stems (*N* = 189) greater than 10 cm of dbh within a 400 ha plot were georeferenced using GPS and genotyped using microsatellite markers (Micheneau et al. 2011) for a previous gene flow study (Angbonda et al. 2021). From September 17th to December 12th, 2016, we collected pods from under-mature trees (*N* = 20), assuming these were mother trees for which we hoped to produce enough seedlings for this study. The sampling of pods was conducted within a maximum radius of 10 m from each focal tree to avoid contamination of our samples by the progeny of other nearby trees. These mother trees (named: S2, S4, S30, S40, S42, S64, S79, S82, S89, S90, S95, S109, S116, S124, S141, S146, S158, S160, S165, and S194) were retained because they were sufficiently isolated from other seed trees to avoid confounding seed sources.

#### Germination in a nursery

Sampled pods were taken to a nursery located on the campus of the Science Faculty of the University of Kisangani where each pod was peeled to extract the seeds. Each pod contained between 1 and 4 seeds, with usually only one viable seed. The latter was placed in a polyethylene bag previously filled with potting soil to allow germination. The soil used came from superficial horizons found at the site.

In the nursery, the experimental setup consisted of six complete randomized blocks; each block contained between 300 and 400 randomized seedlings from different seed carriers, combined with four waves of planting (24–28 Sep, 31 Oct, 01–24 Nov, 06–19 Dec 2016). The planted seedlings were watered regularly to maintain sufficient moisture for germination. After 8 months, two leaflets were collected from the seedlings obtained and carefully dried in silica gel to preserve DNA.

#### Plantation following Nelder design and growth data

Seedlings raised in the nursery were transplanted in October 2017, when they had reached at least 40 cm in height, on three experimental plots in full sun on a land from the concession of a logging company called *Compagnie Forestière et de Transformation* (CFT), located ~9 km from Kisangani (25°15’ 58” E, 0°30’ 42” N, elevation 495 m). The soil consists of old ferralitic layers rich in sand and clay with a pH between 3.5 and 5.5 (Colombaroli et al. 2016), and low fertility (cation exchange capacity - CEC - varies between 2 and 8 meq per 100 g). On each plot, plants were spatially arranged according to one of Nelder’s designs (Nelder 1962), over a circular area of 908 m^2^ as described by Ilunga-Mulala et al. (2021). This method consists of placing 18 plants on each of 12 concentric circles whose radius forms a geometric progression from 0.57 m to 15.57 m, so that the density of plants is very high in the center and decreases towards the edge. We installed three Nelder plots, hereafter referred to as replicates, each containing 180 measured plants labeled with a metal plate and whose growth was monitored for 4 years (up to March 2021). To avoid an edge effect, plants located on the first and last circles were not considered in data analysis. The seedlings transplanted onto the Nelder replicates were from 19 of the 20 maternal origins but only 12 families had at least 10 seedlings among the 540 measured plants, and the most represented family had 81 seedlings. During transplantation, we paid attention that plants from the same maternal origin were well distributed across concentric rings to avoid covariation between potential genetic and competition effects on growth. The height and diameter of seedlings were measured just after their transplantation to record their initial size. The selfed or outcrossed status of seedlings was unknown at the time of transplantation and is thus assumed randomly distributed.

Fifteen trees that died as a result of stress after transplanting were replaced, the last one in August 2018. Growth measurements were taken every three months using a caliper for diameter at 10 cm from the ground and a graduated ruler for total height. The measurements used for the present work are those from March 2018 (5 months after transplantation, to avoid the effect of transplant stress), 2019, 2020 and 2021 (i.e. one year apart). Plants had become saplings at the end of the experiment.

### Genotyping and identification of selfed versus outcrossed seedlings

Total DNA of all seedlings included in the three Nelder replicates was extracted using the NucleoSpin 96 Plant II kit (Macherey-Nagel, Dürren, Germany). We genotyped the seedlings using 11 nuclear microsatellite markers amplified in two multiplexes according to the protocol developed by Micheneau et al. (2011). Paternity analyses were conducted using the neighborhood model implemented in the NMπ software ver. 1.2 (Chybicki and Burczyk 2010) as described by Angbonda et al. (2021), in order to assess for each seedling if it resulted from selfing or outcrossing. We completed the dataset available in Angbonda et al. (2021) by genotyping 250 additional seedlings. In summary, the neighborhood model used the genotypes of adults and seedlings as well as their spatial coordinates to infer the father of each seedling by a maximum likelihood approach, while simultaneously estimating the genotyping error rate at each locus, the selfing rate, the pollen immigration rate and two parameters of a power-exponential pollen dispersal kernel. For further data analyses, we retained only the seedlings for which the probability of being selfed, or outcrossed, was above 0.8.

### Data analyses

Annual growth was calculated by the formula:

1 $$\Delta {\rm{y}}=\frac{\left({y}_{{t}_{2}}-{y}_{{t}_{1}}\right)}{\left({t}_{2}-{t}_{1}\right)}12$$

with Δ*y* the annual height in cm (or diameter in mm) increment, *y* the height (or diameter) measured at successive times *t*_*1*_ and *t*_*2*_ in a month, considering the following measurement times: 5, 17, 29, and 41 months after transplantation. Δ*y* is thus computed at three age intervals, corresponding to plants aged ~2, 3, and 4 years since their germination. Hence, we will refer to the growth of saplings aged 2, 3, or 4 years as the increase in their height or diameter observed, respectively between 5 and 17 months, 17 and 29 months, or 29 and 41 months after transplantation.

At each considered time *t*_*2*_, we estimated the level of competition, *C*, that each plant had experienced from its neighbors (Hegyi 1974; Uhl et al. 2015) as described in Ilunga-Mulala et al. (2021), following the formula:

2 $${C}_{i}={\sum }_{\begin{array}{c}j=1\\ j\ne i\end{array}}^{n}\left(\frac{{D}_{j}}{{D}_{i}{L}_{{ij}}}\right)$$

where *D*_*i*_ and *D*_*j*_ are the respective diameters of subject *i* and competitor *j* and *L*_*ij*_ is the distance between *i* and *j*, and the sum is taken over neighbors within a radius around subject *i* equal to the mean height of plants within the respective Nelder plot.

Above-ground biomass (AGB) 41 months after transplantation was estimated for each plant according to the cone formula:

3 $${\rm{AGB}}=\pi \frac{{(\frac{D}{2})}^{2}H}{3000}\sigma$$

with AGB in kg, *D* the diameter in cm and *H* the height in cm, *π* = 3.1416 and *σ* = 0.64 the wood density of *P. elata* provided by the Global Wood Density Database (Zanne et al. 2009). Although the accuracy of this AGB estimate has not been verified to avoid destructive sampling, it will be used as a proxy of fitness component integrating both height and diameter growth, so that only relative values matter.

#### Modeling growth and assessing the impact of inbreeding

Annual height and diameter increments and AGB were modeled by linear mixed models (LMM). Two main classes of models were tested. The first class (Eq. 4) aimed to compare the percentage of variance explained by the following explanatory variables considered as random factors: replicate (3 categories), family (19 categories corresponding to mother trees), inbreeding (2 categories: outcrossed or selfed, as determined by paternity analysis), age (3 categories) and plant density (10 categories, corresponding the Nelder circle number).

4 $${\Delta {\rm{y}}}_{{ijklmn}}=\mu +{{\rm{\sigma }}}_{i}+{{\rm{\rho }}}_{j}+{\phi }_{k}{+{\alpha }_{l}+{\iota }_{m}+{\chi }_{n}+\varepsilon }_{{ijklmn}}$$

where Δ*y*_*ijklmn*_ is the response variable (annual height or diameter increment) for sapling *i*, in replicate *j*, of family *k*, at age *l*, with inbreeding *m* and in Nelder circle *n*. Parameter *µ* is the model intercept while sapling (*σ*_*i*_), replicate (*ρ*_*j*_), family (*ϕ*_*k*_), age (*a*_*l*_), inbreeding (*ι*_*m*_) and density (χ_*n*_) are random variables and *ε*_*ijklmn*_ is the residual error. Except for *µ*, the other terms follow normal centered distributions whose variances were estimated by fitting the model using the “lmer” function of package “lmerTest” (Kuznetsova et al. 2017).

In the second class of models (Eqs. 6 and 7), while sapling, replicate and family were still treated as random factors, age, inbreeding and competition were treated as fixed effects. Age and inbreeding were still categorical variables, while competition was not treated as a category of plant density but as a quantitative variable depending on the relative sizes of the neighbors: the competition index *C* (Eq. 2). Preliminary analyses showed that the relationship between growth and local competition was well adjusted by a 2nd order polynomial of *C*^1/2^ (lowest AIC, Akaike Information Criterion, when compared to models including only *C* or *C*^1/2^), so that our models involve *C*^1/2^ and *C* among the explanatory variables. We also tested the effect of the initial size at transplantation, *S*, as a covariate. To quantify the impact of ID on growth and its potential interactions with age and competition, the model in Eq. 5 was used.

5 $$\begin{array}{lll}{\Delta {\rm{y}}}_{{ijkl}}={\rm{\mu }}+\,{b}_{1}{S}_{i}+{{\boldsymbol{b}}}_{{\bf{2}}}{A}_{l}+{b}_{3}{I}_{i}+{b}_{4}{C}_{{il}}^{1/2}+{b}_{5}{C}_{{il}}\\\qquad\;\;\;\;\;\;\;+\;{{\boldsymbol{b}}}_{{\bf{6}}}{A}_{l}{I}_{i}+{{{\boldsymbol{b}}}_{{\bf{7}}}{A}_{l}{C}_{{il}}^{1/2}+{{\boldsymbol{b}}}_{{\bf{8}}}{A}_{l}{C}_{{il}}+b}_{9}{I}_{i}{C}_{{il}}^{1/2}+{b}_{10}{I}_{i}{C}_{{il}}\\\qquad\;\;\;\;\;\;\; +\,{{\rm{\sigma }}}_{i}+{{\rm{\rho }}}_{j}+{\phi }_{k}+{\varepsilon }_{{ijkl}}\end{array}$$

It includes as fixed effects (first line): initial size (height or diameter, *S*_*i*_), age (*A*_*l*_), inbreeding (*I*_*i*_ = 0 for selfed sapling, *I*_*i*_ = 1 for outcrossed sapling), competition (*C*_*il*_), as well as (second line) the two-way interaction terms between age, inbreeding and competition, and finally (third line) the random effects of sapling, family and replicate. Parameters *b*_1_ *to b*_10_ are the regression slopes of the fixed effects and interaction terms, with bold values being vectors when terms include a factor with more than two categories (age).

As interaction terms including age were often significant (see results), we also modeled annual height and diameter increment separately for each age (Eq. 6), keeping the same terms as in Eq. (5) except the terms including age and the random factor for sapling (as there was no more repeated measurements). We modeled the AGB at age = 4 years (Eq. 7) as in Eq. (6) but using the cubic root of AGB to approach normality and stabilize the variance of the residuals, and without accounting for initial size as we had found nearly no impact on annual increments. Finally, we modeled the initial height and diameter of seedlings at the time of their transplantation (Eq. 8, where *S*_*ik*_ is the initial height or diameter of individual *i* from family *k*) according to inbreeding (fixed effect) and family (random effect) to assess whether inbreeding depression already occurred in the nursery.

6 $${\Delta {\rm{y}}}_{{ijkl}}={\rm{\mu }}+\,{b}_{1}{S}_{i}+{b}_{2}{I}_{i}+{b}_{3}{C}_{{il}}^{1/2}+{b}_{4}{C}_{{il}}+{b}_{5}{I}_{i}{C}_{{il}}^{1/2}+{b}_{6}{I}_{i}{C}_{{il}}+{{\rm{\rho }}}_{j}+{\phi }_{k}+{\varepsilon }_{{ijk}}$$

7 $${{AGB}}_{{ijk}}^{1/3}={\rm{\mu }}+{b}_{1}{I}_{i}+{b}_{2}{C}_{{il}}^{1/2}+{b}_{3}{C}_{{il}}+{b}_{4}{I}_{i}{C}_{{il}}^{1/2}+{b}_{5}{I}_{i}{C}_{{il}}+{{\rm{\rho }}}_{j}+{\phi }_{k}+{\varepsilon }_{{ijk}}$$

8 $${S}_{{ik}}={\rm{\mu }}+{b}_{1}{I}_{i}+{\phi }_{k}+{\varepsilon }_{{ik}}$$

We estimated the coefficients of variation (CV) of the random factors by the ratio of the standard deviation over the mean of each explanatory variable.

To illustrate graphically the results, we plotted the raw annual height and diameter increments as a function of competition (*C*^1/2^), separately for each year and distinguishing selfed and outcrossed saplings, adjusting a spline function for each case to account for the non-linear effects of competition. We also plotted the mean growth per family according to year, distinguishing again selfed and outcrossed plants. We performed all analyses in the statistical software R version 4.2.1 (R Core Team 2022), using the “ggplot2” (version 3.3.6) package (Wickham 2016) for plotting.

Inbreeding depression was estimated in two ways. First, the level of ID for early growth was estimated from the mean AGB of selfed and outcrossed saplings at 41 months as *δ* = 1–*AGB*_selfed_/*AGB*_outcrossed_. Second, we estimated indirectly *δ* for the lifetime fitness from the comparison of the proportions of selfed seeds, *s*, and selfed adults, *s*’. Assuming that *s* and *s*’ have reached a steady state, that inbreeding is only caused by selfing (no biparental inbreeding), and that ID does not affect the reproduction or mortality of adults (i.e. assuming that ID affects only growth and mortality until adulthood), *s*’ can be predicted from *s* and *δ* as following: *s*’ = *s*(1−*δ*) / (*s*(1-*δ*) + (1−*s*)) = *s*(1−*δ*)/(1-*sδ*), where the *s*(1−*δ*) term is the amount of remaining selfed adults after ID has acted and the (1−*s*) term is the amount of outcrossed adults, which do not suffer from ID (Lande et al. 1994). Hence, by reverting the equation, from estimates of *s* and *s*’, one can estimate *δ* = (*s*−*s*’)/((1−*s*’)*s*). To apply this equation, we used for *s* the proportion of selfed seedlings as assessed by paternity analysis, and for *s*’ the estimated proportion of selfed adults reported by Angbonda et al. (2021) in the parental population, which was based on identity disequilibrium: *s’* = 0.26.

## Results

### Selfing rate

**Fig. 1:**
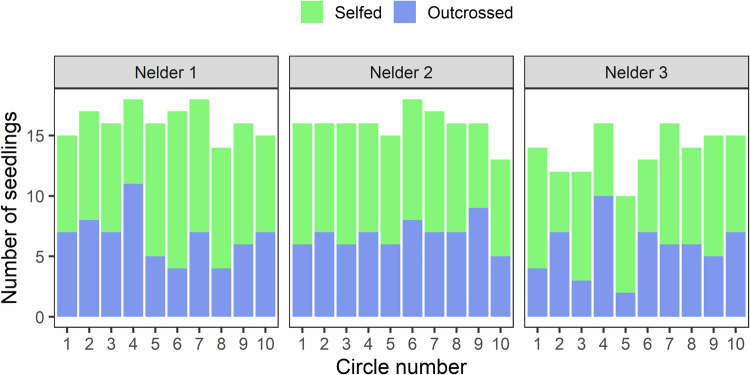
Distribution of the successfully genotyped seedlings unambiguously identified as selfed or outcrossed, by Nelder replicate and circle number. Each circle contained 18 seedlings.

In total, 494 of the 540 measured *P. elata* seedlings were successfully genotyped, among which 267 were selfed, 192 were outcrossed, and 35 remained unassigned (i.e., their genotypes were compatible both with selfing or outcrossing). According to the neighborhood model implemented in NMπ, the self-fertilization rate was estimated at 0.54 ± 0.02, which is close to the proportion of selfed seedlings (58%) if we excluded the unassigned cases. Selfed and outcrossed seedlings were represented in all circles of the three Nelder replicates (Fig. 1).

When considering the 11 mother trees for which the selfed/outcrossed status of at least 12 seedlings was assessed, the selfing rate per seed tree ranged from 44% (family S146) to 85% (family S124), a variation that cannot be explained by chance alone (*X*² test of independence between family and selfing/outcrossing: *p* value < 0.01).

### Survival rate

Among the 540 saplings transplanted in the Nelder plots, only 15 (2.8%) died naturally within the first 41 months of planting and were replaced. Among those, 9 were selfed, 2 were outcrossed, and 4 were undetermined (not genotyped or unassigned). Although the mortality rate seems higher for selfed plants (3.4%) than outcrossed plants (1.0%), Fisher exact test is not significant due to limited sample size (*p* value = 0.13).

### Annual height and diameter increment

Hereafter, we consider the 459 plants for which the selfing or outcrossing status was determined unambiguously. Transplanted seedlings had a mean initial height of 73.5 cm (sd = 29.2, range = 24–160) and mean initial diameter of 7.68 mm (sd = 2.61, range = 3–16). Initial height and diameter did not differ significantly between selfed and outcrossed seedlings (model of Eq. 8; height: *b* = 0.72, *t* = 0.26, *p* value = 0.79; diameter: *b* = 0.357, *t* = 1.49, *p* value = 0.137).

Variance partitioning using the purely random model (Eq. 5, Table 1) shows that plant age explained the highest part of variance in annual height (42.6%) and diameter (31.0%) growth, followed by plant density (Nelder circle number) for diameter growth (22.6%) but not height growth (3.5%). Substantial parts of variance (8.1% to 9.6%) are also due to differences between replicates (Nelder plot). Genetic effects were less important but higher for inbreeding (2.3% to 3.6%) than for family (1.9% to 2.3%), with higher percentages for growth in diameter than in height. The residual variance reached 41.5% and 30.9% for height and diameter growth, respectively (Table 1).

**Table 1:** Percentage of the variability in annual height and diameter increments explained by the factors family (mother tree), density (Nelder circle number), replicate (Nelder plot), age (2, 3 or 4 years old saplings) and inbreeding (outcrossed or selfed saplings), with n the number of categories for each factor.

		Height increment	Diameter increment
	*n*	Variance (%)	Variance (%)
Family	19	1.9	2.3
Density	10	3.5	22.6
Replicate	3	8.1	9.6
Age	3	42.6	31.0
Inbreeding	2	2.3	3.6
Residual		41.5	30.9

The mixed effects models (Eq. 6) show that both height and diameter annual growths were significantly affected by competition, age and inbreeding, with significant interaction between competition and inbreeding effects, and between competition and age effects, but little interaction between age and inbreeding effects (Table 2). The best models integrating these factors explained 64% of the variance in height growth and 79% of the variance in diameter growth. These effects are detailed hereafter.

**Table 2:** Parameter estimates of linear mixed models (*µ* and *b* coefficients of Eq. 5) used to explain annual height and diameter increments of saplings aged 2, 3, or 4 years old.

	Height increment			Diameter increment		
*Fixed effects:*	Estimate	Std. Error	*p* value	Estimate	Std. Error	*p* value
Intercept *µ*	149.90	13.52	**	16.68	1.91	**
Competition *C*^1/2^	26.21	82.84	0.752	−176.45	10.08	***
*C*	−1338.00	87.85	***	−121.00	10.44	***
Age = 3 years *A*_*3*_	−65.13	3.13	***	−6.85	0.30	***
Age = 4 years *A*_*4*_	−83.11	3.13	***	−8.74	0.31	***
Inbreeding *I*_*outcrossed*_	18.86	3.71	***	1.91	0.44	***
Initial size *S*	−0.06	0.04	0.193	0.16	0.07	*
*C*^1/2^ * *I*_*outcrossed*_	−246.40	90.79	**	-45.83	12.67	***
*C* * *I*_*outcrossed*_	−76.30	88.56	0.389	3.17	11.54	0.783
*C*^1/2^ * *A*_*3*_	−873.40	94.53	***	7.52	9.40	0.424
*C* * *A*_*3*_	1270.00	98.74	***	133.36	9.61	***
*C*^1/2^ * *A*_*4*_	−938.60	95.47	***	−33.04	9.74	***
*C* * *A*_*4*_	1397.00	93.39	***	167.74	9.19	***
*A*_*3*_ * *I*_*outcrossed*_	−3.98	4.82	0.409	0.77	0.47	0.102
*A*_*4*_ * *I*_*outcrossed*_	−10.97	4.82	*	−0.37	0.47	0.434
*Random effects:*	S.D.	CV		S.D.	CV	
Sapling	14.31	14.10		2.90	20.79	
Family	8.38	8.26		1.11	7.91	
Replicate	21.94	21.62		3.09	22.13	
Residual error	35.59	35.09		3.46	24.73	

*C* is the Hegyi (1974) competition index; *A*_3_ and *A*_4_ age effects at 3 and 4 years, respectively, relative to 2 years old saplings (i.e. the model assumes b(A_2_) = 0); *I*_outcrossed_ the inbreeding effect for outcrossed saplings relative to selfed saplings (b(*I*_selfed_) = 0); *S* the initial size (height or diameter) at transplantation; S.D. standard deviation; Std. Error standard error; CV the coefficient of variation. Significant relationships are shown (*p < 0.05; **p < 0.01; ***p < 0.001).

**Fig. 2:**
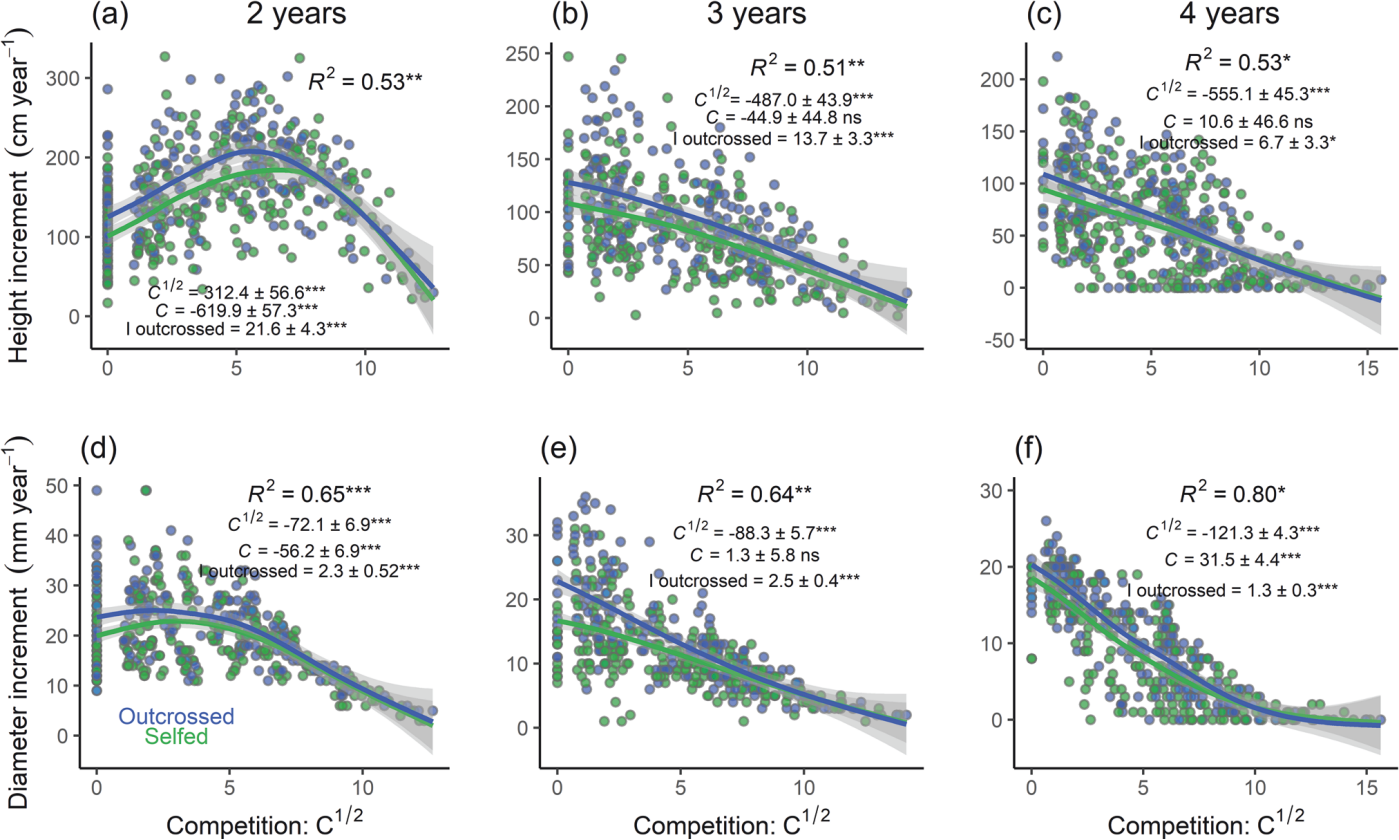
Relationship between height and diameter and level of competition for selfed and outcrossed *Pericopsis elata* saplings at different ages. **a**, **b**, **c** annual height and (**d**, **e**, **f**) diameter increment and local competition, *C*^1/2^, for selfed and outcrossed saplings aged 2 (**a**, **d**), 3 (**b**, **e**) or 4 (**c**, **f**) years old (more specifically, growth from 5 to 17, 17 to 29, or 29 to 41 months after transplantation, respectively). The points are measured data. The shaded areas correspond to the 95% confidence interval of a spline function. Estimated models parameters following Eq. (6) and significant relationships (*p < 0.05; **p < 0.01; ***p < 0.001; ns = not significant) are shown on each panel.

Annual diameter increment was maximal when competition was lowest (up to *c*. 20 mm yr^−1^) and decreased as competition increased, with a nearly linear relationship with the square root of the competition index (*C*^1/2^) for saplings 3 and 4 years old (Fig. 2d–f). By contrast, annual height increment was maximal (up to *c*. 150 cm yr^−1^ for 2 years old samplings) at an intermediate level of competition (*C*^1/2^ around 7; Fig. 2a). Annual increments decreased with age (2, 3 and 4 years saplings) for both height (means of 153.7, 96.6, and 69.1 cm yr^−1^) and diameter (means of 20.0, 12.4 and 9.6 mm yr^−1^) and the growth-competition relationships tended to be less linear with *C*^1/2^ at younger ages (Fig. 2).

Outcrossed individuals showed higher growth rates than selfed individuals for both height (means ± standard deviation of 166.2 ± 59.3 cm yr^−1^and 144.7 ± 58.9 cm yr^−1^; *t* = 4.5; *p* value < 0.001) and diameter (21.3 ± 8.2 mm. yr^-1^ and 19.0 ± 7.5 mm yr^−1^; *t* = 3.4; *p* value < 0.001) for 2 years old saplings, a difference that tended to decay with age but remained statistically significant (Fig. 2). Contrary to our expectations the effect of inbreeding was strongest under low competition, especially for diameter growth, and vanished under the highest competition levels (when *C*^1/2^ > 7), a trend observed across ages (Fig. 2).

**Fig. 3:**
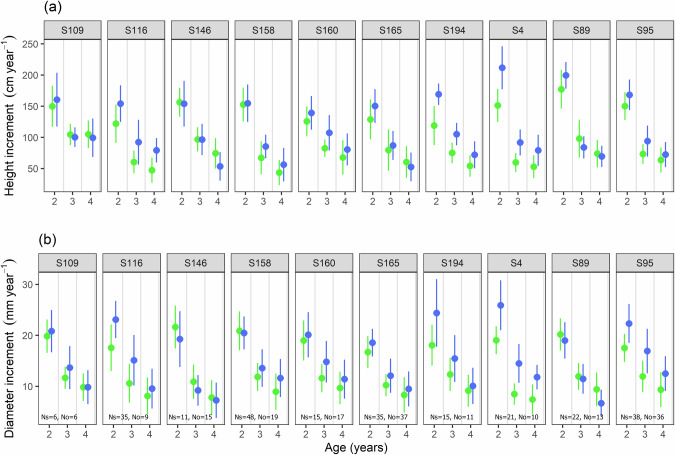
Variation in annual height and diameter of *Pericopsis elata* saplings from 10 families that produced both selfed and outcrossed seedlings. **a** annual height and (**b**) diameter increments. Families consisted of the same maternal origin with selfed (green) and outcrossed (blue) saplings (at least 6 of each), according to age (2, 3 or 4 years old saplings). The mother number is indicated on top of each plot. Ns and No are the number of selfed and outcrossed individuals, respectively. The dots are the means with standard deviations.

Among the ten families including at least six selfed and six outcrossed progeny (Fig. 3), we observe that growth was consistently higher for outcrossed than selfed plants in five families (mother trees n° S4, S95, S116, S160, S194) and nearly so in two additional ones (n° S158, S165), while there was no difference for one of them (n° S109) and even a slight trend of higher performances of selfed plants in two families (n° S89, S146). However, for the latter two families, selfed progeny appeared more often in the low density Nelder circles than outcrossed progeny, explaining the reverse pattern, while selfed and outcrossed progeny were well balanced across the density gradient of the Nelder plots in the other families.

### Estimation of inbreeding depression

**Fig. 4:**
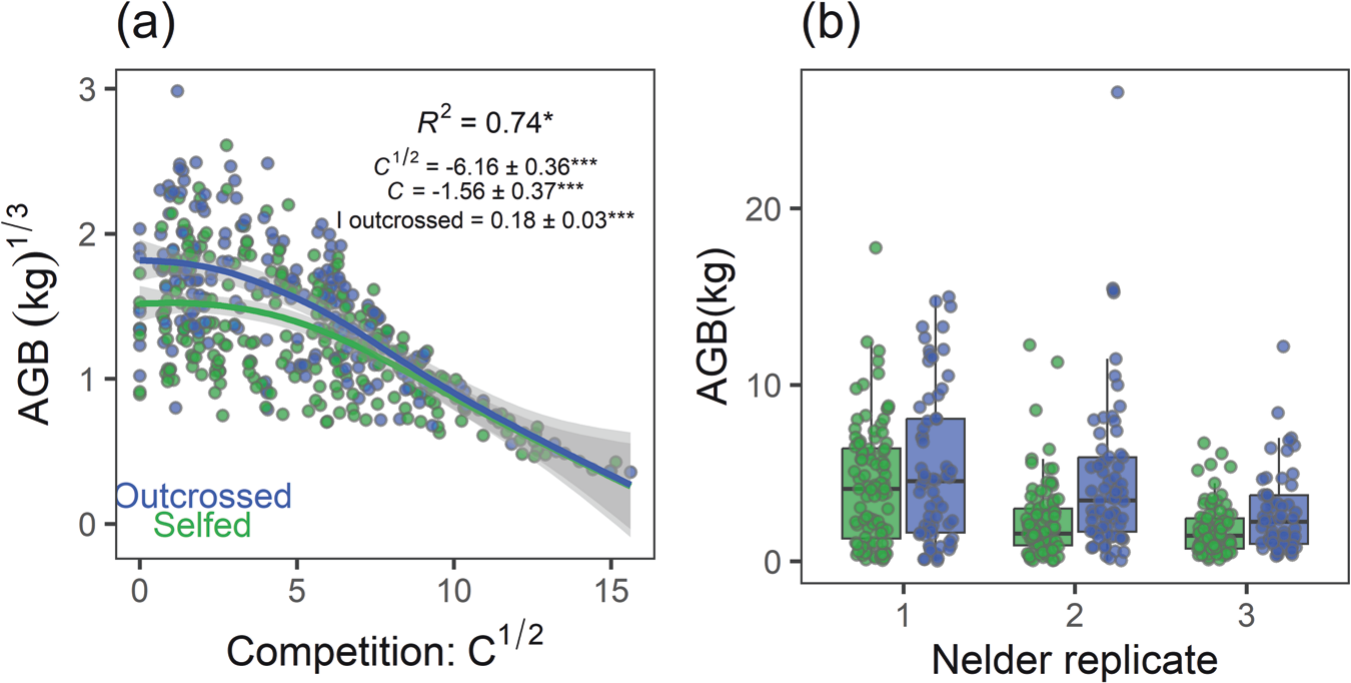
Distribution of above-ground biomass (AGB) of selfed and outcrossed *Pericopsis elata* saplings accros competition level and Nelder replicates. **a** local competition, and (**b**) Nelder replicate. The points are measured data 41 months after transplantation for outcrossed (blue) and selfed (green) individuals. The curves in (**a**) are smoothed using the “gam” method, where the shaded areas correspond to the 95% confidence interval. Model parameters (Eq. 7) and significant relationships (*p < 0.05; **p < 0.01; ***p < 0.001) are shown in graph (**a**).

Outcrossed saplings had higher AGB (4.3 ± 4.0 kg, mean ± standard deviation) than selfed saplings (2.9 ± 2.8 kg), leading to an ID of this fitness component of *δ* = 0.33 (i.e., the AGB of selfed saplings is 33% lower than that of outcrossed saplings). However, the difference between selfed and outcrossed saplings was well marked when the level of competition was low (*δ* = 0.39 when *C*^1/2^ < 4) but disappeared under high competition (*δ* ≈ 0 when *C*^1/2^ > 10; Fig. 4a). All Nelder replicates showed this difference (Fig. 4b). Therefore, both the outcrossing effect and the interaction between outcrossing and *C*^1/2^ appeared significant in the statistical model, which explains 73.8% of variance (Table 3). The relationship between AGB and *C*^1/2^ is nonlinear but was well fitted with a 2nd order polynomial.

**Table 3:** Parameters and properties of a linear mixed model (Eq. 7) used to explain the above-ground biomass (AGB) of *Pericopsis elata* saplings, 41 months after transplantation, according to local competition (C, considering a 2nd order polynomial of C^1/2^), inbreeding (selfed or outcrossed) and the interaction between these explanatory variables.

*Fixed effects:*	Estimate	Std. Error	*p* value
Intercept *µ*	1.26	0.17	*
Competition *C*^1/2^	−6.16	0.36	***
*C*	−1.56	0.37	***
Inbreeding *I*_*outcrossed*_	0.18	0.03	***
*C*^1/2^ * *I*_*outcrossed*_	−2.18	0.58	***
*C* * *I*_*outcrossed*_	0.09	0.58	0.880
*Random effects:*	S.D.	CV (%)	
Family	0.09	2.51	
Replicate	0.29	8.36	
Residual error	0.28	7.99	

S.D. standard deviation, Std. Error standard error and CV the coefficient of variation. Significant relationships are shown (*p < 0.05; **p < 0.01; ***p < 0.001).

The lifetime ID estimated from the decay of selfing rate between the seedling stage (*s* = 0.54) and the adult stage (*s*’ = 0.26 for adults of the source population; Angbonda et al. 2021), led to *δ* = 0.70 (i.e., the survival rate until adulthood of selfed seedlings is 70% lower than that of outcrossed seedlings).

## Discussion

Understanding the effects of inbreeding on tropical rainforest tree species is of direct practical importance for genetic conservation, breeding, and environmental restoration to guide sustainable management of populations, particularly for threatened species. Relatively few studies have directly measured ID in tropical tree populations (Chaves et al. 2011; Jolivet et al. 2013; Bessega et al. 2017; Pupin et al. 2019; Aguiar et al. 2020; Takeuchi and Diway 2021), one obvious reason being the difficulty to measure all fitness components in long-living organisms. For our study, we collected seeds of *P. elata* from known mothers in a natural forest and planted them following a Nelder design to compare the behavior of plants resulting from selfing with those resulting from outcrossing after monitoring growth during 41 month.

### High and variable primary self-fertilization rate in *P. elata*

*Pericopsis elata* is an exceptional model due to its high proportion of selfed individuals: 0.54 ± 0.02 among seedlings, 0.26 ± 0.10 among adults in the studied population (Angbonda et al. 2021). By comparison, other tropical trees generally show a rate of self-fertilization <10% (Ward et al. 2005; Dick et al. 2008). Another tropical tree with similar selfing rate is the Asiatic *Rubroshorea acuminata* (Dyer) P.S.Ashton & J.Heck. (Naito et al. 2008), and high selfing rates were also reported in *Eucalyptus* species (e.g. Byrne 2008; Griffin et al. 2019), which include tropical species native to the Australian continent. Among African tropical trees, fairly high rates of selfing were also reported in *Erythrophleum suaveolens* (Guill. & Perr.) Brenan (c. 16%; Duminil et al. 2016b) and *Baillonella toxisperma* Pierre (c. 30%; Duminil et al. 2016a) based on seed or seedling genotypes but there was no evidence of selfed adults.

We observed significant variation in primary selfing rate among *P. elata* families, ranging from 0.44 to 0.85. The origin of this variation is unknown but it could result from variation in the availability of surrounding flowering conspecific trees if it was mediated by pollen limitation (Naito et al. 2008; Tani et al. 2009), and/or from variation in morphological or physiological traits in flowers, which could have then a genetic basis. Given that the median distance between mates of outcrossing events reached about 260 m while each mother tree was surrounded by several tens of adults within this radius in the studied population (Angbonda et al. 2021), pollen limitation is probably not a limiting factor. If genetic factors were involved, it offers the possibility to select mother trees producing a higher proportion of outcrossed seeds for plantations or plant breeding.

### Despite substantial inbreeding depression, a portion of selfed *P. elata* do survive and reproduce

Inbreeding decays with age in *P. elata* (inbreeding coefficient *F* = 0.3 in seeds for *F* = 0.15 in adults; Angbonda et al. 2021), as documented in most tropical trees producing a portion of selfed progeny (Cloutier et al. 2007; Doligez and Joly 1997; Gaino et al. 2010; Hufford and Hamrick 2003). This is an expected manifestation of ID in species where a portion of juveniles are inbred, for example due to a mixed mating system (Hufford and Hamrick 2003).

Our experimental results confirm that ID affects the growth of *P. elata* seedlings, particularly under low competition conditions (Table 2; Figs. 2, 3). ID often manifests in the traits of tree species (e.g., Tambarussi et al. 2017). For example, the size, diameter at breast height (dbh) and volume of outcrossed progeny of *Eucalyptus regnans* F. Muell. at 45 months of age were on average 11%, 18% and 37% greater, respectively, than those of the selfed progeny (Griffin and Cotterill 1988). However, in *P. elata* we did not observe ID on growth in the nursery. In addition, we did not detect a significant effect of ID on survival rate but the mortality rate was probably too low in our experiment to be able to detect such an effect.

### After environmental factors, the growth of *P. elata* seedlings is more affected by inbreeding effect than family effect

Annual diameter or height growth of *P. elata* seedlings varied substantially among years, across the density gradient and across replicates of Nelder devices, reflecting the impact of different environmental factors that each explained usually between 8 and 40% of the variation in growth (Table 1). By comparison, a much smaller portion of the variance in annual growth was explained by family (1.9–2.3%) or inbreeding (2.3–3.6%) effects. Nevertheless, the impact of these genetic factors is certainly not negligible in the lifetime of trees because they act constantly while environmental factors can fluctuate substantially over time. The family effect implies that growth ability has a heritable component, indicating potential for improvement in tree breeding. However, as consanguinity has a stronger impact than family, ID is a major genetic factor to take into account.

### Inbreeding depression manifests itself during the growth of *P. elata* trees

As often done with self-compatible organisms, we estimated ID from the relative loss of fitness of selfed individuals compared to outcrossed ones (*δ* = 0 in the absence of ID while *δ* = 1 if selfed progeny die before reaching adulthood or cannot reproduce). So, based on estimates of the above-ground biomass (AGB) of selfed and outcrossed saplings 41 months after transplantation, ID was estimated as *δ* = 0.33, and up to *δ* = 0.39 under low intra-specific competition. AGB is only a proxy of a fitness component expressed here at an early stage. The actual level of ID for lifetime reproductive success might be larger, for example if the initial growth disadvantage of selfed trees tends to worsen over time because faster growing neighbors increasingly compete for resources (Cheptou et al. 2001), or if ID also affects seed set independently of tree size, or germination rate.

Estimating *δ* for all fitness components of trees is very laborious (but see Griffin et al. 2019). However, under specific assumptions, in particular when the sole source of inbreeding is selfing and that ID affects the survival until adulthood but not the reproductive success of adults, a lifetime *δ* can be estimated from the decay of the proportion of selfed individuals between the seedling stage (*s*) and the adult stage (*s*’). In the adult population used in this study and described in Angbonda et al. (2021), individual heterozygosity was not correlated with tree diameter, nor with their reproductive success assessed from the parentage analysis (unpublished results), so that all or most of the ID might be expressed during the seedling and sapling stages (lower growth and survival of selfed individuals), allowing us to estimate the lifetime ID from *s* and *s*’. This led to *δ* = 0.70. In other words, the survival rate of selfed juveniles until adulthood is 70% lower than the one of outcrossed juveniles. This lifetime ID estimate is considerably higher than the one based on AGB. This should not be surprising as AGB at 41 months is only one of several fitness components but, in any case, the *δ* derived from *s* and *s*’ reflects ID in natural conditions while the one derived from AGB concerns the experimental conditions of a plantation in full light, so that the two estimates are not directly comparable.

A literature survey of *δ* estimates in 58 plant species (Winn et al. 2011b) concluded that the lifetime ID averaged *δ* = 0.51 for species showing a mixed mating system (selfing rate between 20 and 80%), and the only reported tree species with a mixed mating system, *E. regnans* (Griffin et al. 2019), had *δ* = 0.80, close to the value estimated for *P. elata*. Nevertheless, long-term follow up of ID in *Eucalyptus* species usually found that selfed individuals did not survive until the adult stage (Griffin et al. 2019; Nickolas et al. 2019), contrary to what is found in *P. elata*. The long-term maintenance of a mixed mating system in these conditions remains unexplained because deleterious recessive mutations should be purged efficiently. A model potentially explaining the maintenance of a mixed mating system, called selective interference (Lande et al. 1994), occurs when many deleterious recessive alleles are generated by mutation so that few, if any, selfed individuals can eventually reproduce. However, given that nearly a quarter of reproducing adults result from selfing events in *P. elata*, this model cannot explain the maintenance of its mixed mating system. We hypothesize that reproductive assurance, the capacity to reproduce under low pollinator or mate availability, might have been a strong enough selective force in favor of selfing in *P. elata*, counter balancing the selection of mechanisms favoring outcrossing in response to ID (Ruan and da Silva 2012).

### Inbreeding depression is maximal at low plantation density in *P. elata*

Meta-analyses show that ID tends to be stronger under stressful conditions (Armbruster and Reed 2005 ; Fox and Reed 2010) and/or under higher intraspecific competition (e.g., Yun and Agrawal 2014), especially when outcrossed and selfed individuals compete. ID expressed at the seedling or sapling stage in *P. elata* does not fit this pattern as it was maximal under low density and seemed to vanish under high competition (Figs. 2, 4a), resulting in significant interaction terms between competition and inbreeding when modeling growth (Table 2). ID also did not appear in the nursery, where plant roots had access to a limited volume of earth (about 2 l), limiting plant growth after several months. As *P. elata* is a light-demanding tree, which does not survive a long time in the shade of a closed canopy at the seedling or young sapling stage, only juveniles lucky enough to be in a large forest gap have a chance to grow fast enough to reach a reasonable size and eventually reproduce (Ouédraogo et al. 2014; Umunay et al. 2017). In light of our experimental results, we hypothesize that it is in such favorable conditions that ID acts most against selfed juveniles.

Contrary to plantation density, seedling age showed no or low interaction with inbreeding when modeling growth (Table 2), and ID was visible in all the families where selfed and outcrossed seedlings were well distributed across the density gradient (Fig. 3). It should be noted that our results obtained in a controlled environment may not reflect what happens in natural conditions, so that the impact of ID on seedling growth and survival in natural forests would deserve further studies.

### Implications for the sustainable management of *P. elata* stands

This study has practical implications for genetic resource conservation and management of *P. elata* populations, including planting. Given the observed ID in *P. elata*, selecting early outcrossed progeny would be an advantage. However, ID was not detectable in the nursery; hence applying an early selection of faster-growing seedlings at this stage does not seem efficient to select the outcrossed ones. Alternatively, we could select seed trees that deliver more outcrossed seeds for planting projects, as we observed variation in outcrossing rate among maternal families, although none of the family tested produced more than 56% outcrossed seeds. Genotyping seedlings at an early stage to identify the outcrossed ones could also be considered but with a SSR genotyping cost of 5–10 USD per sample using current technologies, it might be too expensive to be economically viable. Moreover, monospecific plantations are usually subject to natural thinning so that we expect a progressive elimination of slow-growing selfed trees and it is not guaranteed that an early elimination of selfed seedlings would necessarily benefit the final wood production. Therefore, optimizing the environmental conditions for plantations probably remains the priority for silviculture (Ilunga-Mulala et al. 2024).

Given that we observed a significant family effect on growth rate, which is thus heritable, proper recording of the mother trees in plantations would be helpful to identify the best families (for growth rate and other traits related to disease or stress resistance, or conformation) and later select the best-performing seed trees for new plantations.

As recommended in technical handbooks (e.g. Daïnou et al. 2021), seed collection sources must be well selected to maintain the regeneration potential of tree species. For plantations aiming at restoring degraded forests or enriching forest gaps, where natural generation is projected in the future, it is essential to keep a large genetic diversity of mother trees, otherwise one would favor ID in the next generation due to consanguineous mating, even for outcrossed progeny (Bittencourt and Sebbenn 2008; Angbonda et al. 2021). To reduce the risk of using and establishing inbred plants and to guarantee sufficient genetic diversity, a few measures can be considered when planting *P. elata*: (*i*) collect seeds on a large number of mother trees, sufficiently spaced given that nearby trees tend to be closely related (Angbonda et al. 2021); (ii) keep track of the maternal origin of seedlings in order to control the mixture of different origins and balance their respective contributions in plantations. In logged forests where remnant populations of *P. elata* may be subject to increasing regeneration failure due to inbreeding depression resulting from higher selfing or mating between related individuals (Cascante et al. 2002; Fukue et al. 2007; Naito et al. 2008; Tani et al. 2009), leaving sufficient well-spaced seed trees after logging should be considered to maintain a regeneration potential of *P. elata* (Angbonda et al. 2021), and enriching forest gaps with nursery-raised *P. elata* seedlings can compensate for the deficit of natural regeneration (Ouédraogo et al. 2014).

### Data archiving

The data used in this study are available from the Dryad Digital Repository: 10.5061/dryad.xwdbrv1nv.
